# An improved *in vitro* 3T3-L1 adipocyte model of inflammation and insulin resistance

**DOI:** 10.1080/21623945.2024.2414919

**Published:** 2024-10-17

**Authors:** Ifeoluwa A. Odeniyi, Bulbul Ahmed, Benjamin Anbiah, Grace Hester, Peter T. Abraham, Elizabeth A. Lipke, Michael W. Greene

**Affiliations:** a Department of Nutritional Sciences, Auburn University, Auburn, AL, USA; b Department of Chemical Engineering, Auburn University, Auburn, AL, USA

**Keywords:** Adipocyte, inflammation, insulin resistance, in vitro model, 3T3-L1 cells

## Abstract

Tumor necrosis factor alpha (TNF-α)/hypoxia-treated 3T3-L1 adipocytes have been used to model inflamed and insulin-resistant adipose tissue: this study examines gaps in the model. We tested whether modulating TNF-α/hypoxia treatment time could reduce cell death while still inducing inflammation and insulin resistance. Adipocytes were treated with TNF-α (12 h or 24 h) and incubated in a hypoxic chamber for 24 h. To examine maintenance of the phenotype over time, glucose and FBS were added at 24 h post initiation of treatment, and the cells were maintained for an additional 48 h. Untreated adipocytes were used as a control. Viability, insulin resistance, and inflammation were assessed using Live/Dead staining, RT-qPCR, ELISA, and glucose uptake assays. Treatment for 12 h with TNF-α in the presence of hypoxia resulted in an increase in the percentage of live cells compared to 24 h treated cells. Importantly, insulin resistance and inflammation were still induced in the 12 h treated adipocytes: the expression of the insulin sensitive and inflammatory genes was decreased and increased, respectively. In 72 h treated adipocytes, no significant differences were found in the viability, glucose uptake or insulin-sensitive and inflammatory gene expression. This study provides a modified approach to in vitro odeling adipocyte inflammation and insulin resistance.

## Introduction

Obesity is a growing public health problem worldwide. In the United States, the obesity epidemic is of concern because approximately 39.8% of adults are considered obese [1]. Obesity is linked to insulin resistance and inflammation which are thought to increase the risk of developing non-communicable diseases such as certain forms of cancer, type 2 diabetes, hypertension, and cardiovascular diseases [2].

Adipose tissue dysfunction leads to modulation of humoral factor expression, including adipokines that contribute to obesity-linked pathophysiological changes in the body [3]. Some of the best-described adipokines that are upregulated include tumour necrosis factor alpha (TNF-α), interleukin 6 (IL-6), and C-C Motif Chemokine Ligand 2 (CCL2, as known as MCP-1), while adiponectin is down regulated. Adipose insulin resistance is characterized by an impaired response to insulin, which results in reduced glucose transporter 4 (GLUT4)-mediated glucose uptake, glycogen synthesis, and dysregulated lipolysis, leading to enhanced release of free fatty acids [4].

Of the many *in vitro* models of adipose tissue [5], the 3T3-L1 fibroblast cell line is one of the most commonly used *in vitro* models to study adipocyte differentiation and biology because there are well-established protocols for inducing the fibroblasts to become adipocytes which synthesize and accumulate triglyceride [6]. Differentiated 3T3-L1 adipocytes are highly insulin sensitive. However, numerous factors associated with the obese state have the capacity to induce insulin resistance in differentiated 3T3-L1 adipocytes including TNF-α, IL-6, dexamethasone, high insulin, glucosamine, and exposure to hypoxic environment [7]. Hypoxia has been demonstrated to potentiate the inflammatory effect of TNF-α [8]. Consistent with this finding, Lo et al. demonstrated that a combined 24 h treatment of hypoxia and TNF-α induced insulin resistance in differentiated 3T3-L1 adipocytes *in vitro*, and importantly, most accurately mimicked transcriptional changes in the adipose tissue of diet-induced obese insulin-resistant mice [9].

Co-culture of adipocytes and numerous cells such as hepatocytes, macrophages, and cancer cells often require a long-term culture. Long-term *in vitro* models of insulin resistant adipocytes with concurrent inflammation have relied on treatment with single factors such as IL-1β, IL-6, and TNF-α, [10–13] which do not mimic *in vivo* insulin-resistant adipose tissue [9]. Yet, it is not known whether the hypoxia and TNF-α model of insulin resistance and inflammation could be maintained long term in culture. Therefore, we undertook a study to establish the hypoxia and TNF-α *in vitro* model of insulin resistance and inflammation for long-term culture. Our first aim was to confirm the use of TNF-α and hypoxia to induce acute insulin resistance and inflammation in differentiated 3T3-L1 adipocytes and examine cell viability in the model. Our second aim was to investigate the ability of TNF-α and hypoxia treatment to establish a long-term model of adipocyte insulin resistance and inflammation, which would allow for the investigation of metabolic cross-talk between obesity-related insulin resistant and inflammatory adipocytes and other cells (e.g. cancer cells, monocytes, myocytes, or hepatocytes) in a co-culture system.

## Methods and materials

### Reagents

3T3-L1 preadipocytes were purchased from the American Type Culture Collection (ATCC, Manassas, VA, USA). Dulbecco’s Modified Eagle’s Medium (DMEM), glutamine, and penicillin-streptomycin (P/S) were obtained from Gibco (ThermoFisher Scientific, Grand Island, NY, US). Trypsin/EDTA and foetal bovine serum (FBS) were obtained from LONZA (Walkersville, MD, US) and Atlanta Biologicals (Lawrenceville, GA, US), respectively. Clear tissue culture dishes and plates were obtained from Corning (Corning, NY, US). Black 24-well tissue culture plates were obtained from (Azenta US, Inc., Indianapolis, IN,). Insulin was from Roche (Mannheim, Germany). 3-Isobutyl-1-methylxanthine (IBMX), dexamethasone, cytochalasin B, and rosiglitazone were from Sigma (St Louis, MO, US). 2-(*N*-(7-Nitrobenz-2-oxa-1,3-diazol-4-yl)Amino)-2-Deoxyglucose (2-NBDG) and the Pierce BCA protein assay kit were purchased from ThermoFisher Scientific (Rockford, IL, US). Primary antibodies AKT and phosphoAKT (ser473) were purchased from Cell Signaling Technology (Beverly, MA, US). Secondary antibody goat anti-mouse IgG IRDye 680RD and goat anti-rabbit IgG IRDye 680RD were obtained from LI-COR (Lincoln, NE, US). Total RNA isolation Qiazol reagent was purchased from Qiagen (Hilden Germany). Reverse transcription SuperScript IV First-Strand Synthesis System and Power SYBR Green PCR Mastermix were purchased from ThermoFisher Scientific (Waltham, MA, US). Murine primers to the *eukaryotic translation elongation factor 2 (Eef2)*, *solute carrier family 2 member 4 (Slc2A4, aka GLUT4), Adiponectin (Adipoq), C-C Motif Chemokine Ligand 2 (Ccl2) and Interleukin 6 (IL6)* genes were purchased from Integrated DNA Technologies, Inc. (Coralville, IA, US). Phosphate-buffered saline (PBS) and 2-propanol were from Fisher Scientific (Pittsburgh, PA, US).

### 3T3-L1 Cell Culture and differentiation to obtain adipocytes

3T3-L1 preadipocytes were cultured in Growth Media (high glucose (4.5 g/L) DMEM containing 10% FBS and 1X P/S. Cells were subcultured prior to 80% confluence in a 10 cm plate. To differentiate the 3T3-L1 preadipocytes, cells were seeded in 6-well or 24-well plates and allowed to grow to approximately 120% confluence in Growth Media, then Differentiation Media containing 10 μg/mL insulin, 1 μM rosiglitazone, 1 μM dexamethasone, and 0.5 mm IBMX was added to each well (Day 0 of differentiation). After 3 days, the Differentiation Media was removed and replaced with Maintenance Media (Growth Media plus 5 μg/mL insulin). Maintenance Media was used to feed the cells at Day 5 and 7 after the initiation of differentiation. Finally, Maintenance Media was replaced with Growth Media at Day 9.

### 3T3-L1 adipocyte treatments

To induce insulin resistance and inflammation, adipocytes (Day 10 after initiation of differentiation) were incubated in low glucose (1 g/L) DMEM containing 0.1% BSA and 20 ng/mL recombinant rat TNF-α (kindly provided by Eugene Holowachuk and Mary Ruhoff [14]). The cells were placed in a hypoxia chamber (STEMCELL Technologies, Cambridge, MA, US) and allowed to equilibrate with 1% O_2_ & 5% CO_2_ for 5 min prior to sealing the chamber and being placed in a 37°C incubator for 12 or 24 h. In the 12 h TNF-α treated cells, the cells were removed from the hypoxia chamber, the treatment media was replaced with low glucose (1 g/L) DMEM containing 0.1% BSA media, and then the cells were placed back in the hypoxia chamber and allowed to equilibrate with 1% O_2_ & 5% CO_2_ for 5 min prior to sealing the chamber and being placed in a 37°C incubator for 12 h. Control 3T3-L1 adipocytes were incubated in low glucose (1 g/L) DMEM containing 0.1% BSA media under normoxic conditions for 24 h. For the long-term insulin resistance and inflammation model, the cells were removed from the hypoxia chamber and 4.5 g/L glucose and 10% FBS was added to each well. As a control for the long-term insulin resistance and inflammation model, 3T3-L1 adipocytes were incubated in low glucose (1 g/L) DMEM containing 0.1% BSA media under normoxic conditions for 24 h, and then 4.5 g/L glucose and 10% FBS were added to each well.

### Immunoblotting

Adipocytes were collected and RIPA buffer added, and they were sonicated for 30 s at 5-s intervals (5 sec sonication and 5 sec on ice). The Pierce BCA protein assay kit was used to measure the concentration of protein. Before each Western blot, the same amount of protein samples was heated for 10 min at 70°C. Using 10% polyacrylamide gel electrophoresis at 100 mA for two hours, the protein in the samples was separated and then transferred to nitrocellulose membranes at 100 mA for two hours. The membrane was blocked for an hour with phosphate-buffered saline (TBST) with 5% skim milk. After that, the membrane was kept at 4°C for one night with the primary antibody and then at room temperature for one hour with the secondary antibody. A Li-COR blot scanner was used to analyse the membrane. The ImageStudio software was used to measure the intensity of the bands (LI-COR Biosciences, NE, US).

### Gene expression: RNA isolation and rt-qPCR

Adipocytes were harvested from the well plates using Qiazol lysis reagent (Qiagen, Hilden Germany) and store at −80°C prior to RNA isolation. RNA was extracted using the Qiagen RNA mini kit (Qiagen, Hilden, Germany) according to the manufacturer’s instructions, from three independent experiments of biological duplicates. Yield was quantified at 260 nm and purity assessed by 260/280 nm ratio using the Nanodrop 1000 Spectrophotometer (Thermo). All samples were stored at −20°C until further use. For first strand cDNA synthesis, 1 µg of total RNA was reverse transcribed using the SuperScript IV Reverse Transcriptase (SSIV, Thermo Fisher Scientific, Waltham, MA, USA) in a reaction volume of 20 µl following the manufacturer’s instructions. The final samples of cDNA synthesized were divided and stored in aliquots at −20°C until further analysis.

**Figure 1.**
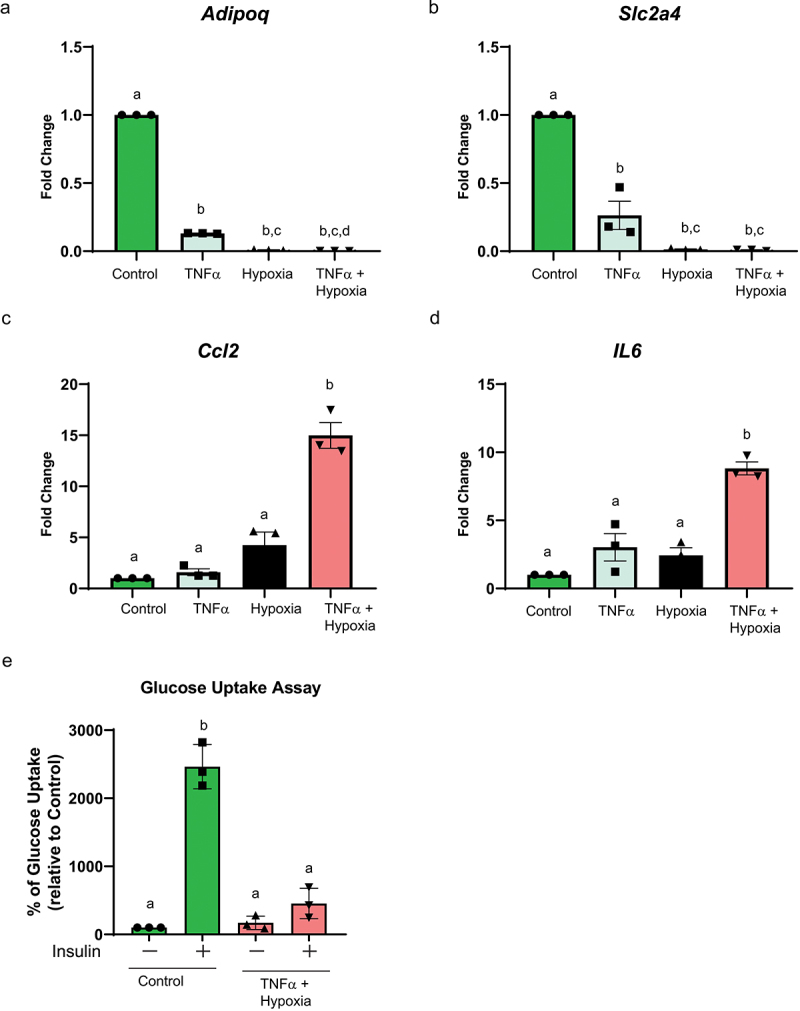
Tnf-α and hypoxia downregulated *insulin sensitivity gene expression, upregulated inflammatory gene expression, and* inhibited insulin-stimulated glucose uptake: mature differentiated 3T3-L1 adipocytes were treated with tnf-α and/or hypoxia for 24 h. Insulin sensitive gene markers *adipoq (a)* and *Slc2a4 (b), and* inflammation gene markers *Ccl2* (c) and *IL6* (d) and were assessed using rt-qPCR. gene expression was normalized using *Eef2* as reference gene, and the δδct method was used to assess fold changes (*n* = 3 independent experiments). (e) mature differentiated 3T3-L1 adipocytes were treated with tnf-α and hypoxia for 24 h. Glucose uptake was assessed using a glucose analog (2-NBDG). Cells were stimulated with or without 100 nM insulin for 20 min. Cytochalasin B (cytB) treated cells were used to assess non-specific glucose uptake. The non-specific transport values obtained from the cytB group were subtracted from the values obtained from the cells stimulated with or without insulin (*n* = 3 independent experiments). The data is presented as the percent of the non-stimulated control. Data was analysed using one-way ANOVA with Tukey post-hoc test (A-E). Bar = mean ± SE. Means that do not share same letter for each treatment are significantly different (*p* < 0.05).

**Figure 2.**
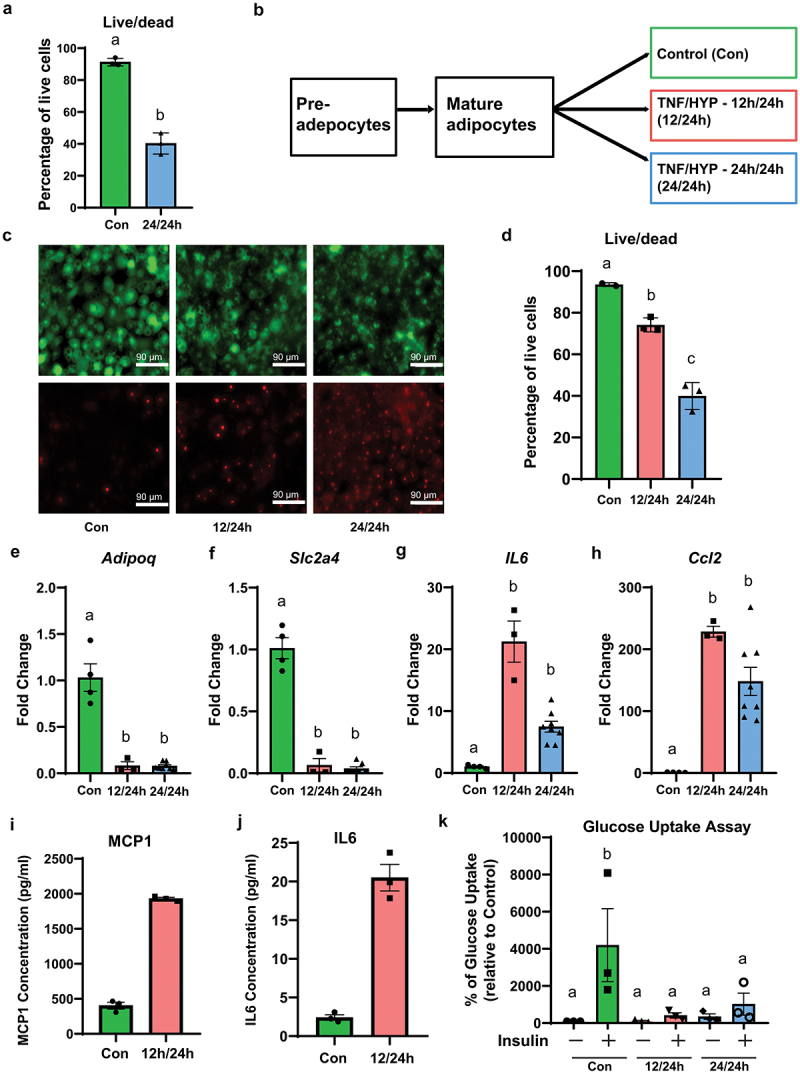
Modulating the treatment time with tnf-α in the presence of hypoxia increased the viability of cells in adipocyte insulin resistance model yet maintained the inflammatory and insulin resistant phenotype. Mature differentiated 3T3-L1 adipocytes were treated with tnf-α and hypoxia for 24 h or with tnf-α and hypoxia for 12 h and 24 h, respectively. (a) Quantitation of live/dead staining shown as the percentage of live cells. (b) Flow chart to test the effect of modulating the treatment time of tnf-α in the presence of hypoxia on cell death and makers of inflammation and insulin resistance. (c) Representative fluorescence images of live (green) and dead (red) cells in the control, tnf-α/Hypoxia treated for 12 h/24 h (TNF/HYP, 12 h/24 h) and the tnf-α/Hypoxia treated for 24 h/24 h (TNF/HYP, 24 h/24 h) groups. Images were taken at 20X magnification. (d) Quantitation of live/dead staining shown as the percentage of live cells (*n* = 3 independent experiments). (E-H) rt-qPCR to assess insulin sensitive *and* inflammation gene markers. Insulin sensitive gene markers *adipoq (E)* and *Slc2a4 (F), and* inflammation gene markers *IL6* (G) and *Ccl2* (H) are shown. Gene expression was normalized using *Eef2* as reference gene, and the δδct method was used to assess fold changes (*n* = 3 independent experiments). (I-J) conditioned media was pooled from 3 replicate wells and assessed for secreted inflammation molecules using murine specific ELISAs at 24 h post treatment for MCP1 (I) and IL-6 (J). Data was analyzed using a t-test (I-J). (K) Glucose uptake was assessed using a glucose analog (2-NBDG). Cells were stimulated with or without 100 nM insulin for 20 min. Cytochalasin B (cytB) treated cells were used to assess nonspecific glucose uptake. The nonspecific transport values obtained from the cytB group were subtracted from the values obtained from the cells stimulated with or without insulin (*n* = 3 independent experiments). The data is presented as the percent of the non-stimulated control. Data was analyzed using one-way ANOVA with Tukey post-hoc test (D-K). Bar = mean ± SE. Means that do not share same letter for each treatment are significantly different (*p* < 0.05).

**Figure 3.**
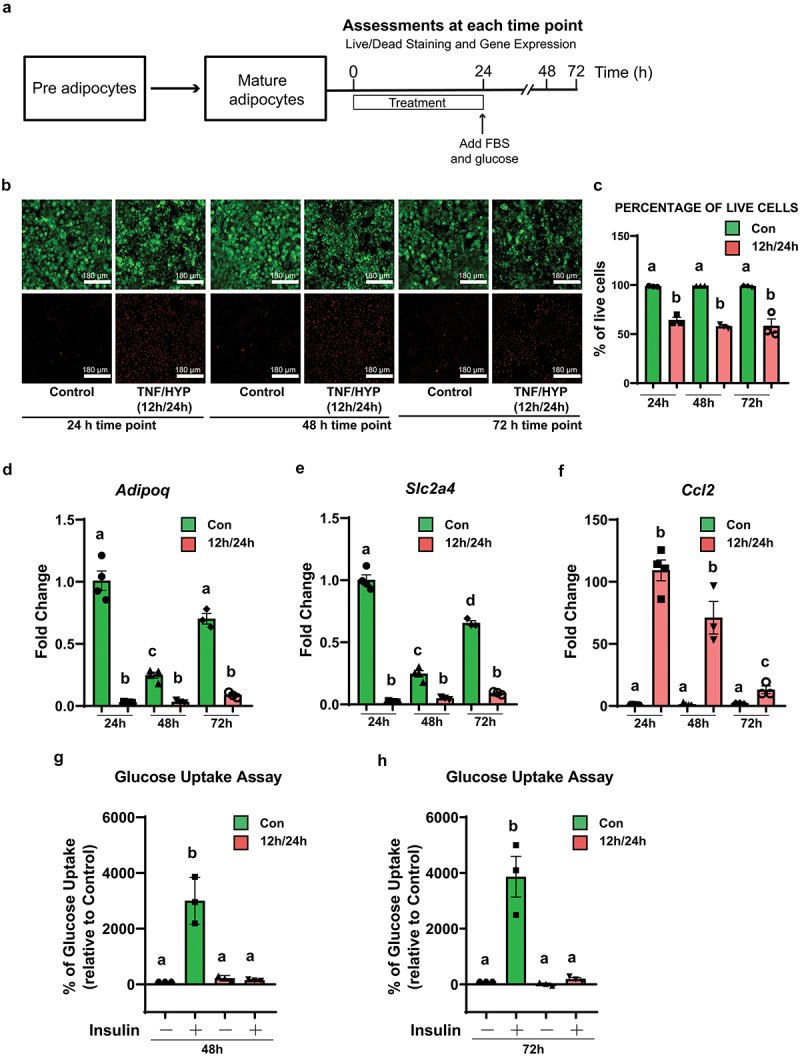
Tnf-α (12 h) and hypoxia (24 h) induce long-term insulin resistance in an *in vitro* adipocyte insulin resistance model. (a) Mature differentiated 3T3-L1 adipocytes were treated with tnf-α for 12 h and hypoxia for 24 h to create the insulin resistance group and the untreated cells served as a control. After 24 h, 4.5 g/L glucose and 10% FBS were added to both the treated and control adipocytes and monitored at 48 and 72 h post tnf-α/Hypoxia treatment. (b) Representative fluorescence images of live (green) and dead (red) cells in the control group versus the tnf-α/Hypoxia treated for 12 h/24 h at different time points. Images were taken at 10X magnification. (c) Cell viability was assessed by live/dead staining at 24 h, 48 h, and 72 h (*n* = 3 independent experiments). Insulin sensitive markers *adipoq (d)*, *Slc2a4* (e) and inflammatory marker *Ccl2* (f) were assessed using rt-qPCR at different time points. Gene expression was normalized using *Eef2* as reference genes, and the δδct method was used to assess fold changes (*n* = 3 independent experiments). (g-h) glucose uptake was assessed using a glucose analog (2-NBDG) in control and 48 h treated cells (g) and control and 72 h treated cells (H). Cells were stimulated with or without 100 nM insulin for 20 min. Cytochalasin B (cytB) treated cells were used to assess nonspecific glucose uptake. The nonspecific transport values obtained from the cytB group were subtracted from the values obtained from the cells stimulated with or without insulin (*n* = 3 independent experiments). The data is presented as the percent of the non-stimulated control. Data was analyzed using one-way ANOVA with Tukey post-hoc test (C-H). Bar = mean ± SE. Means that do not share same letter for each treatment are significantly different (*p* < 0.05).

Expression of mRNA was quantified using reverse transcription-quantitative polymerase chain reaction (RT-qPCR). The relative expression of target genes mRNA was compared using the comparative ΔΔCT method. The genes of interest were *SLC2A4* (*aka GLUT4*), *Adiponectin (Adipoq)*, *Ccl2* and *IL6*. For the gene expression data presented in Figure 1, qPCR was performed using the RT2 SYBR Green qPCR Mastermix (Qiagen) using a MyIQ Real-Time PCR system (Bio-Rad, Hercules, CA). For the gene expression data presented in Figures 2–3, the Power SYBR Green PCR Master Mix (Thermo Fisher Scientific, Waltham, MA, USA) was used. Primer pairs targeting specific genes were used at a concentration of 0.2 µM to each reaction. RT-qPCR was performed at the following conditions: polymerase activation (95°C for 2 min); 40 cycles of PCR were performed, each lasting 15 s at 95°C and 1 min at 60°C, followed by a melting curve lasting 15 s at 95°C, 1 min at 60°C and 1 s at 95°C. Each RT-qPCR reaction’s specificity of its final product was verified using melting curve analysis. Reactions were carried out in triplicate using 1 µl of 1:4 diluted cDNA in a 20 µl final reaction volume in a Quant Studio 3 Real-Time PCR System (ThermoFisher Scientific, Waltham, MA, USA). The threshold cycle (Ct) values were used to calculate relative gene expression, using the ΔΔCT method, with *EEF2* as house-keeping gene. All the oligonucleotides were made by Integrated DNA Technologies, and primer sequences of all the genes investigated in this study, including the housekeeping gene, are outlined in Supplemental Table 1.

### Live/Dead staining

To test the viability of the treated adipocytes over time, the LIVE/DEAD® Viability/Cytotoxicity Kit (ThermoFisher Scientific, Waltham, MA, USA) was used to assess cell viability in accordance with the manufacturer’s protocol. A viability solution comprising 2 M calcein AM, 4 mm ethidium homodimer-1 (EthD-1) and 20 µg/mL Hoechst 33,342 (Calbiochem) was added to the wells to label live cells, dead cells, and every cell nucleus. Using an Echo Revolve microscope (Echo, San Diego, CA, US), images of fluorescently labelled cells were captured. ImageJ (version 2.0.0-rc-68/1.52e) was used to calculate cell viability. The number of cell nuclei and the number of dead cells were manually counted, and the viability % was determined using equation 1. The cell viability assay was analysed statistically using two-way ANOVA followed by the Tukey post hoc test.

(1) $$\mathrm{Viability}\left(\%\right)=\frac{\mathrm{No}.\mathrm{of}\;\mathrm{cell}\;\mathrm{nuclei}-\mathrm{No}.\mathrm{of}\;\mathrm{dead}\;\mathrm{cells}}{\mathrm{No}.\mathrm{of}\;\mathrm{cell}\;\mathrm{nuclei}}$$

### Assessment of tnf-α MCP1, and IL-6 by ELISA

Recombinant rat TNF-α levels in the condition media obtained from the treated adipocytes media were measured using a rat TNF-α Quantikine ELISA kit according to the manufacturer’s protocol (R&D Systems Inc.). Secreted MCP-1 and IL-6 levels in the condition media obtained from the adipocytes were measured using murine MCP-1 and IL-6 Quantikine ELISA kits, respectively, according to the manufacturer’s protocol (R&D Systems Inc.).

### Glucose uptake assay (in vitro 2-NBDG assay)

To prepare for the glucose uptake assay, 3T3-L1 cells were differentiated into matured adipocytes in 24-well black plates. Cells were then incubated with experimental media containing 1 g/L glucose and reduced or serum-free conditions for 24 h prior to the assay. For the cytochalasin B (cytB) group, the culture medium was discarded, and cells were washed twice with glucose-free, serum-free medium. Cells were then stimulated with 100 nM of insulin. The culture medium was discarded from all wells except the cytB group, and cells were washed twice with glucose-free medium, with a ten-minute incubation during the second rinse to ensure complete glucose removal. Subsequently, the fluorescently labelled deoxyglucose analog 2-NBDG stock solution was diluted to 100 μM with glucose-free, serum-free medium and was added to the plate which was then incubated for 20 min in a 5% CO2 incubator at 37°C. Following incubation, the cells were washed three times with 200 μl/well of ice-cold PBS to prevent 2-NBDG efflux. Fluorescence was measured using a microplate reader (λex 467 nm, λem 542 nm, and non-specific transport values obtained from the cytB group were subtracted from the values obtained from the cells stimulated with or without insulin. The data is presented as the percent of the non-stimulated control.

### Statistical analysis

All experiments were run at least in triplicate. After checking for normal distribution, data were analysed using one-way ANOVA with Tukey post-hoc test. P-values less than 0.05 were determined to be significant.

## Results

### Tnf-α and hypoxia induce insulin resistance in vitro

To confirm the previously reported *in vitro* adipocyte model of acute insulin resistance and inflammation [9], differentiated adipocytes were treated with TNF-α, hypoxia, or a combined treatment of TNF-α and hypoxia for 24 h in low glucose (1.0 g/L) serum-free media containing 0.1% BSA. We found that 20 ng/mL (1.2 nM) TNF-α treatment alone significantly (*p* < 0.001) decreased the expression of insulin-sensitive genes *Adipoq* and *Slc2a4 (Glut4)* by the fold change 0.13 ± 0.0 and 0.27 ± 0.1, respectively. In the case of hypoxia, the expression of insulin-sensitive genes *Adipoq* and *Slc2a4 (Glut4)* (*p* < 0.001) significantly decreased by the fold change 0.01 ± 0.0 and 0.01 ± 0.0 respectively. When the adipocytes were treated with the combined TNF-α and hypoxia treatment, the expression of *Adipoq* and *Slc2a4 (Glut4)* were significantly (*p* < 0.001) reduced (fold change 0.00 ± 0.0 and 0.01 ± 0.0 respectively). This combined treatment of TNF-α and hypoxia reduced the expression of *Adipoq* and *Slc2a4 (Glut4)* to the greatest degree of the three treatments tested (Figures 1a & b). We also assessed pro-inflammatory markers associated with insulin resistance; we did not find any significant difference in *Ccl2* and *IL6* expression when differentiated adipocytes were treated with individual TNF-α and hypoxia treatments. However, the combined TNF-α and hypoxia treatment significantly (*p* < 0.001) increased *Ccl2* and *IL6* by the fold change of 14.98 ± 1.25 and 8.82 ± 0.47, respectively (Figures 1c & d). To further assess the insulin-resistant phenotype of the adipocytes, after 24 h treatment with combined TNF-α and hypoxia, cells were stimulated with 100 nM of insulin for 20 min and fluorescently labelled 2-NBDG was added to assess the insulin stimulated glucose uptake. We found that insulin stimulated the control cells by (2.60-fold) relative to the unstimulated control cells. However, there was no significant difference in the insulin stimulation of glucose uptake in the adipocytes treated with TNF-α and hypoxia for 24 h in the presence or absence of insulin relative to the control (Fig. IE). To further assess the insulin-resistant phenotype of the adipocytes, after 24 h of treatment with combined TNF-α and hypoxia, cells were induced by 50 nM insulin for 15 min. AKT activation – a critical node in insulin signalling [15] - was assessed by western blot using an antibody that recognizes Ser 473 phosphorylation [16]. We found that phosphorylated AKT expression significantly (*p* < 0.001) decreased in adipocytes treated with TNF-α and hypoxia compared to untreated adipocytes (Suppl. Fig. 1). Taken together, we confirm that TNF-α and hypoxia acutely inhibit insulin signalling *in vitro*.

### Altering TNF- α treatment time improves cell viability in the adipocyte insulin resistance model

We next sought to determine whether treatment with 1.2 nM TNF-α and hypoxia for 24 h was cytotoxic to the adipocytes. As shown in Figure 2a, the 24 h TNF-α/24 h hypoxia treatment significantly reduced (*p* < 0.001) the percentage of live adipocytes. This finding led us to question whether the recombinant TNF-α persisted in the treatment media over the course of the 24 h treatment period. Therefore, we assessed the TNF-α concentration in the media over the course of 24 h. As shown in Supplemental Figure 2, we observed very low recombinant rat TNF-α immunoreactivity over 24 h in the control. In the 24 h TNF-α/24 h hypoxia treatment group, we observed high (*p* < 0.001) levels of recombinant rat TNF-α concentration in the media, and the recombinant rat TNF-α concentration was maintained over the course of 24 h (T0 = 2882.5 ± 46.8, T12 = 2548.4 ± 301.3 & T24 = 2826.0 ± 223.0 compared to the control cell media).

To examine whether reducing the TNF-α treatment time to 12 h by replacing the media with fresh media to remove the recombinant TNF-α improved cell viability, we tested the effect of a 24 h vs 12 h TNF-α treatment in the presence of hypoxia for 24 h (Figure 2b). As shown in Figure 2c, and D, we observed that the pronounced cell death associated with the 24 h TNF-α/hypoxia treatment was reduced when the TNF-α treatment time was reduced to 12 h. A significant increase (*p* < 0.001) was observed in the percentage of live cells in the 12 h TNF- α/24 h hypoxia treatment (74.2 ± 2.4) compared to the 24 h TNF-α/24 h hypoxia treatment (40.0 ± 4.6) (Figure 2c). As expected, when the treatment media was replaced with fresh media in the 12 h TNF-α/24 h hypoxia group, we found that the recombinant TNF-α concentration was significantly reduced (T12 = 240.4 ± 109.1 & T24 = 189.8 ± 34.8 compared to the control cell media).

We next assessed whether reducing the TNF-α treatment time regulated the expression of the insulin-sensitive genes *Adipoq* and *Slc2a4 (Glut4)* to a similar extent to that of the 24 h TNF-α/24 h hypoxia treatment. Similar to the adipocytes treated with 24 h TNF-α/24 h hypoxia, the adipocytes treated with 12 h TNF-α/24 h hypoxia resulted in a significant reduction in *Adipoq* (0.08 ± 0.1-fold) and *Slc2a4 (Glut4)* (0.07 ± 0.1-fold) expression compared to the untreated cells (*p* = 0.001) (Figures 2e & f). Further, no significant differences were found between the 12 h and 24 h TNF-α treatment time in both markers of insulin responsiveness (Figures 2e & f). Pro-inflammatory markers *Ccl2* and *IL6* expression were also assessed. Consistent with the markers of insulin sensitivity, no significant difference was found between the adipocytes treated either for 12 h or 24 h with TNF-α, while the expression of the inflammatory genes *IL6* (21 ± 4-fold) and *Ccl2* (228 ± 11-fold) were significantly increased (*p* < 0.001) compared to the untreated cells (Figures 2g & h). To confirm that the 12 h TNF-α/24 h hypoxia treatment resulted in secretion of proinflammatory molecules, we examined the accumulation of MCP1 and IL-6 in the conditioned media pooled at 24 h post treatment. Conditioned media at 24 h from adipocytes treated with 12 h TNF-α/24 h hypoxia resulted in a significant increase in MCP1 (1930 ± 19.9 pg/mL) and IL-6 (20.5 ± 1.7 pg/mL) presence compared to untreated cells MCP1 (402.2 ± 48.2 pg/mL) and IL-6 (2.4 ± 0.4 pg/mL) (*p* < 0.001), respectively (Figures 2i & j).

To directly assess insulin action we examined insulin stimulated glucose uptake level between the control cells and the treated cells in the 24 h vs 12 h TNF-α treatment in the presence of hypoxia for 24 h. There was no significant difference in the insulin stimulated glucose uptake in both the treated groups, and it was significantly different from the control cells in the presence of insulin (Figure 2k).

Taken together, our findings indicate that reducing the treatment time of TNF-α to 12 h by changing the culture media removes the recombinant TNF-α used for the treatment and improves cell viability while maintaining the inflammatory/insulin resistant phenotype.

### Adipocytes maintained long-term insulin resistant phenotype in vitro

To determine whether the 12 h TNF-α/24 h hypoxia model of adipocyte inflammation/insulin resistance could be maintained long-term, post-treatment we added glucose and FBS to the treated cells to achieve a level of glucose (2.5 mm) and FBS (10%) that is used in the adipocyte maintenance media and then examined cell viability and assessed the expression of inflammation and insulin sensitivity markers for an additional 48 h (72 h of total time) (Figure 3a). The viability of the cells in the untreated group was not significantly different at the 24 h (98.6 ± 0.4), 48 h (99.1 ± 0.0) and 72 h (98.8 ± 0.7) time points post treatment (Figures 3b & c). No significant difference was also found in the viability of the 12 h TNF-α/24 h hypoxia treated cells across the different time points: 24 h (64.0 ± 3.7), 48 h (57.6 ± 1.6) and 72 h (58.2 ± 8.8) (Figures 3b & c).

We observed that both the insulin-sensitive genes *Adipoq* and *Slc2a4 (Glut4)* significantly (*p* = 0.001 and *p* = 0.001, respectively) decreased by the fold changes of 0.03 ± 0.0 and 0.02 ± 0.0, respectively, at the 24 h time point, and the downregulation was maintained at the 48 and 72 h time points (Figures 3d & e). On the other hand, the pro-inflammatory gene *Ccl2* significantly (*p* < 0.001) increased by the fold change of 109.2 ± 9.6 at the 24 h time point. Even though *Ccl2* expression was not significantly different at the 48 h time point, the fold increase was reduced to 13.05 ± 3.82 at the 72 h time point (Figure 3f). To further confirm whether the 12 h TNF-α/24 h hypoxia model of adipocyte inflammation/insulin resistance could be maintained as a long term model, we assessed the insulin stimulated glucose uptake at 48 h and 72 h. The 12 h TNF-α/24 h hypoxia treated adipocytes failed to respond to insulin at both the 48 h (Figure 3g) and 72 h (Figure 3g) time points. Taken together, our findings indicate that the 12 h TNF-α/24 h hypoxia treatment of adipocytes results in an adipocyte inflammation/insulin resistance model that can be maintained an additional 48 h in culture after treatment with no further detrimental effect on cell viability.

## Discussion

The 3T3-L1 adipocyte model is widely used to study insulin resistance and inflammation. The effect of hypoxic conditions on adipocytes and adipose tissues has been extensively studied [7,17–20]. *In vitro* exposure to hypoxia for 24 h has been demonstrated to induce adipocyte dysfunction which was further characterized by the modulation of at least 1346 genes [18], while the *in vitro* hypoxia treatment of adipocytes for 16 h has been shown to rapidly and robustly impact the inhibition of insulin signalling through IRS-1 serine phosphorylation [20]. TNF-α is also widely used to induce insulin resistance [11,21,22]. In addition, TNF-α is able to upregulate genes related to chemotaxis such as *Ccl2, Ccl7*, and *Ccl9* in adipose tissue [7]. Chemotaxic proteins attract macrophages to infiltrate in the obese adipose tissue, resulting in chronic inflammation [23]. In the current study, TNF-α and hypoxia were used to develop an insulin resistance phenotype in differentiated 3T3-L1 adipocytes. Glucose uptake assays confirmed the impaired insulin action. Our study demonstrated that the combinatory treatment of 24 h TNF-α and 24 h hypoxia induced inflammation and insulin resistance in differentiated adipocytes *in vitro* to a significantly greater extent than single treatment using TNF-α or hypoxia alone. Glucose uptake assays confirmed that insulin action was impaired in the 24 h TNF-α and 24 h hypoxia treated adipocytes. This finding is supported by the earlier report by Lo et al. in which the combinatory treatment of 24 h TNF-α and 24 h hypoxia most mimicked the inflamed adipose tissue in diet-induced obese mice [9]. Consistent with Lo et al. [9], we observed that a reduction in insulin-stimulated AKT serine phosphorylation was associated with downregulation *GLUT4* and *adiponectin* gene expression and an upregulation of *Ccl2* and *IL6* gene expression.

The cytotoxic effects of TNF-α treatment in adipocytes are dose dependent [24]. TNF-α can also induce a dedifferentiated state when cells are treated with greater than 5 nM [25]. Using 1.2 nM recombinant TNF-α, we found that the 24 h TNF-α and 24 h hypoxia treatment caused significant cell death to the adipocytes. Excessive cell death in the model is a confounding variable if the inflammatory/insulin resistant adipocytes are to be used in co-culture experiments. Another limitation of the previously reported model for use in co-culture experiments is that we observed that the recombinant TNF-α used to induce inflammation and insulin resistance remained in the media and, as such, would confound the response of the co-cultured cells to humoral factors secreted by the inflammatory/insulin resistant adipocytes. Thus, we examined whether altering the treatment time with TNF-α by switching the media to fresh media removed the recombinant TNF-α and improved cell viability. We found out that treating the adipocytes with TNF-α for 12 h instead of 24 h removed the recombinant TNF-α and improved the viability of the cells. To examine the insulin-resistant phenotype, glucose uptake assays confirmed the impaired insulin action in the 12 h TNF-α and 24 h hypoxia treated adipocytes. To further examine the phenotype, we determined whether the treated adipocytes secreted inflammatory proteins. Our results showed an increased concentration of both CCL2 and IL-6 secreted into the media in our 12 h TNF-α and 24 h hypoxia treatment group. Thus, our 12 h TNF-α and 24 h hypoxia treatment maintained the inflammatory and insulin-resistant phenotype in the adipocytes.

To examine whether the inflammatory and insulin resistance state could be maintained beyond 24 h, we treated adipocytes with 12 h TNF-α and 24 h hypoxia and then provided the treated cells with glucose and FBS. We then assessed gene expression of insulin resistance and inflammation markers after treatment and 24 h and 48 h post treatment. We found no significant differences in the downregulation of *adipoq* and *Slaca4* (GLUT4) gene expression in the 12 h TNF-α/24 h hypoxia treated cells across the different time points. In contrast, *Ccl2* expression increased at 24 h and was maintained at 48 h but was significantly decreased at 72 h. However, at all post-treatment time points, *Ccl2* was significantly increased compared to insulin-sensitive control adipocytes.

The differentiated 3T3-L1 pre-adipocyte model has provided valuable insights into obesity-related disease conditions. However, a limitation is that they may not fully reflect the complexity and heterogeneity of human adipose tissue. Despite our efforts to refine an obese insulin resistance *in vitro* model, the phenotype of the model remains severe. The downregulation of *adipoq* and *Slaca4* (GLUT4) expression in the model is more severe compared to reductions observed in insulin-resistant humans [26,27]. Further studies may be required to evaluate the model in comparison to *in vivo* obese insulin resistance conditions. In conclusion, this study provides a modified approach to modelling inflammation and insulin resistance in adipocytes *in vitro* which has the capacity to be used to examine obesity-linked insulin resistance and inflammation in a co-culture system.

## Supplementary Material

Supplemental Material

## Data Availability

The data that support the findings of this study are available from the corresponding author, [M.W.G.], upon reasonable request.

## References

[1] Hales Cm CM, Fryar CD, Ogden CL. Prevalence of obesity among adults and youth: united States, 2015-2016. In: NCHS data brief no 288(Hyattsville. (MD): National Center for Health Statistics); 2017. p. 1–11. 29155689

[2] Ellulu MS, Patimah I, Khaza’ai H, et al. Obesity and inflammation: the linking mechanism and the complications. Arch Med Sci. 2017;13(4):851–863. doi: 10.5114/aoms.2016.58928 28721154 PMC5507106

[3] Feijoo-Bandin S, Aragon-Herrera A, Morana-Fernandez S, et al. Adipokines and inflammation: focus on cardiovascular diseases. Int J Mol Sci. 2020;21(20):7711. doi: 10.3390/ijms21207711 33081064 PMC7589803

[4] Kahn BB, Flier JS. Obesity and insulin resistance. J Clin Invest. 2000;106(4):473–481. doi: 10.1172/JCI10842 10953022 PMC380258

[5] Dufau J, Shen JX, Couchet M, et al. In vitro and ex vivo models of adipocytes. Am J Physiol Cell Physiol. 2021;320(5):C822–C841. 33439778 10.1152/ajpcell.00519.2020

[6] Knutson VP, Balba Y. 3T3-L1 adipocytes as a cell culture model of insulin resistance. Vitro Cell Dev Biol Anim. 1997;33(2):77–81. doi: 10.1007/s11626-997-0025-2 9081212

[7] Long TJ, Takeno M, Sprenger CC, et al. Capillary force seeding of sphere-templated hydrogels for tissue-engineered prostate cancer xenografts. Tissue Eng Part C Methods. 2013;19(9):738–744. doi: 10.1089/ten.tec.2012.0388 23373788 PMC3719465

[8] Bhattacharya I, Domínguez AP, Drägert K, et al. Hypoxia potentiates tumor necrosis factor-α induced expression of inducible nitric oxide synthase and cyclooxygenase-2 in white and brown adipocytes. Biochem Biophys Res Commun. 2015;461(2):287–292. doi: 10.1016/j.bbrc.2015.04.020 25881506

[9] Lo KA, Labadorf A, Kennedy NJ, et al. Analysis of in vitro insulin-resistance models and their physiological relevance to in vivo diet-induced adipose insulin resistance. Cell Rep. 2013;5(1):259–270. doi: 10.1016/j.celrep.2013.08.039 24095730 PMC3874466

[10] Lagathu C, Yvan-Charvet L, Bastard JP, et al. Long-term treatment with interleukin-1β induces insulin resistance in murine and human adipocytes. Diabetologia. 2006;49(9):2162–2173. doi: 10.1007/s00125-006-0335-z 16865359

[11] Stephens JM, Lee J, Pilch PF. Tumor necrosis factor-alpha-induced insulin resistance in 3T3-L1 adipocytes is accompanied by a loss of insulin receptor substrate-1 and GLUT4 expression without a loss of insulin receptor-mediated signal transduction. J Biol Chem. 1997;272(2):971–976. doi: 10.1074/jbc.272.2.971 8995390

[12] Lagathu C, Bastard J-P, Auclair M, et al. Chronic interleukin-6 (IL-6) treatment increased IL-6 secretion and induced insulin resistance in adipocyte: prevention by rosiglitazone. Biochem Biophys Res Commun. 2003;311(2):372–379. doi: 10.1016/j.bbrc.2003.10.013 14592424

[13] Kang S, Tsai LT, Zhou Y, et al. Identification of nuclear hormone receptor pathways causing insulin resistance by transcriptional and epigenomic analysis. Nat Cell Biol. 2015;17(1):44–56. doi: 10.1038/ncb3080 25503565 PMC4281178

[14] Holowachuk EW, Ruhoff MS. Restoration of abated T cell stimulation activity of mature dendritic cells. Biochem Biophys Res Commun. 2001;285(3):594–597. doi: 10.1006/bbrc.2001.5213 11453633

[15] Taniguchi CM, Emanuelli B, Kahn CR. Critical nodes in signalling pathways: insights into insulin action. Nat Rev Mol Cell Biol. 2006;7(2):85–96. doi: 10.1038/nrm1837 16493415

[16] Bayascas JR, Alessi DR. Regulation of Akt/pkb Ser473 phosphorylation. Mol Cell. 2005;18(2):143–145. doi: 10.1016/j.molcel.2005.03.020 15837416

[17] Halberg N, Khan T, Trujillo ME, et al. Hypoxia-inducible Factor 1α induces fibrosis and insulin resistance in white adipose tissue. Mol Cell Biol. 2009;29(16):4467–4483. doi: 10.1128/MCB.00192-09 19546236 PMC2725728

[18] Mazzatti D, Lim FL, O’Hara A, et al. A microarray analysis of the hypoxia-induced modulation of gene expression in human adipocytes. Arch Physiol Biochem. 2012;118(3):112–120. doi: 10.3109/13813455.2012.654611 22352407

[19] Yin J, Gao Z, He Q, et al. Role of hypoxia in obesity-induced disorders of glucose and lipid metabolism in adipose tissue. Am J Physiol Endocrinol Metab. 2009;296(2):E333–42. doi: 10.1152/ajpendo.90760.2008 19066318 PMC2645021

[20] Regazzetti C, Peraldi P, Gremeaux T, et al. Hypoxia decreases insulin signaling pathways in adipocytes. Diabetes. 2009;58(1):95–103. doi: 10.2337/db08-0457 18984735 PMC2606898

[21] Ruan H, Hacohen N, Golub TR, et al. Tumor necrosis factor-alpha suppresses adipocyte-specific genes and activates expression of preadipocyte genes in 3T3-L1 adipocytes: nuclear factor-kappaB activation by tnf-alpha is obligatory. Diabetes. 2002;51(5):1319–1336. doi: 10.2337/diabetes.51.5.1319 11978627

[22] Qi C, Pekala PH. Tumor necrosis factor-alpha-induced insulin resistance in adipocytes. Proc Soc Exp Biol And Med Soc Exp Biol Med (New Y, N.Y.). 2000;223(2):128–135. doi: 10.1046/j.1525-1373.2000.22318.x 10654615

[23] Weisberg SP, McCann D, Desai M, et al. Obesity is associated with macrophage accumulation in adipose tissue. J Clin Invest. 2003;112(12):1796–1808. doi: 10.1172/JCI200319246 14679176 PMC296995

[24] Prins JB, Niesler CU, Winterford CM, et al. Tumor necrosis factor-α induces apoptosis of human adipose cells. Diabetes. 1997;46(12):1939–1944. doi: 10.2337/diab.46.12.1939 9392477

[25] Xing H, Northrop JP, Grove JR, et al. TNFα-mediated inhibition and reversal of adipocyte differentiation is accompanied by suppressed expression of PPARγ without effects on pref-1 expression. Endocrinology. 1997;138(7):2776–2783. doi: 10.1210/endo.138.7.5242 9202217

[26] Kern PA, Di Gregorio GB, Lu T, et al. Adiponectin expression from human adipose tissue: relation to obesity, insulin resistance, and tumor necrosis factor-α expression. Diabetes. 2003;52(7):1779–1785. doi: 10.2337/diabetes.52.7.1779 12829646

[27] Marette A, Maurie`ge P, Marcotte B, et al. Regional variation in adipose tissue insulin action and GLUT4 glucose transporter expression in severely obese premenopausal women. Diabetologia. 1997;40(5):590–598. doi: 10.1007/s001250050720 9165229

