# Supplementary material for: An improved in vitro 3T3-L1 adipocyte model of inflammation and insulin resistance
Source: Adipocyte. 2024 Oct 17;13(1):2414919. doi: 10.1080/21623945.2024.2414919 (PMC11487959; doi:10.1080/21623945.2024.2414919)
Supplement: Supplemental Material [file KADI_A_2414919_SM0933.zip › Suppl Table 1.docx]

Supplemental Table 1

| **Gene** |  | **Sequence (5′-3′)** |
| --- | --- | --- |
| *Ccl2* (MCP1) | Forward | caagatgatcccaatgagtag |
|  | Reverse | ttggtgacaaaaactacagc |
| tnfa | Forward | ctatgtctcagcctcttctc |
|  | Reverse | catttgggaacttctcatcc |
| *Adipoq* (Adiponectin) | Forward | ccactttctcctcatttctg |
|  | Reverse | ctagctcttcagttgtagtaac |
| *IL6* | Forward | aagaaatgatggatgctacc |
|  | Reverse | gagtttctgtatctctctgaag |
| *Eef2* | Forward | agaacatatattgctggcg |
|  | Reverse | caacagggttagatttcttg |
