# Supplementary figures and images for: An improved in vitro 3T3-L1 adipocyte model of inflammation and insulin resistance
Source: Adipocyte. 2024 Oct 17;13(1):2414919. doi: 10.1080/21623945.2024.2414919 (PMC11487959; doi:10.1080/21623945.2024.2414919)

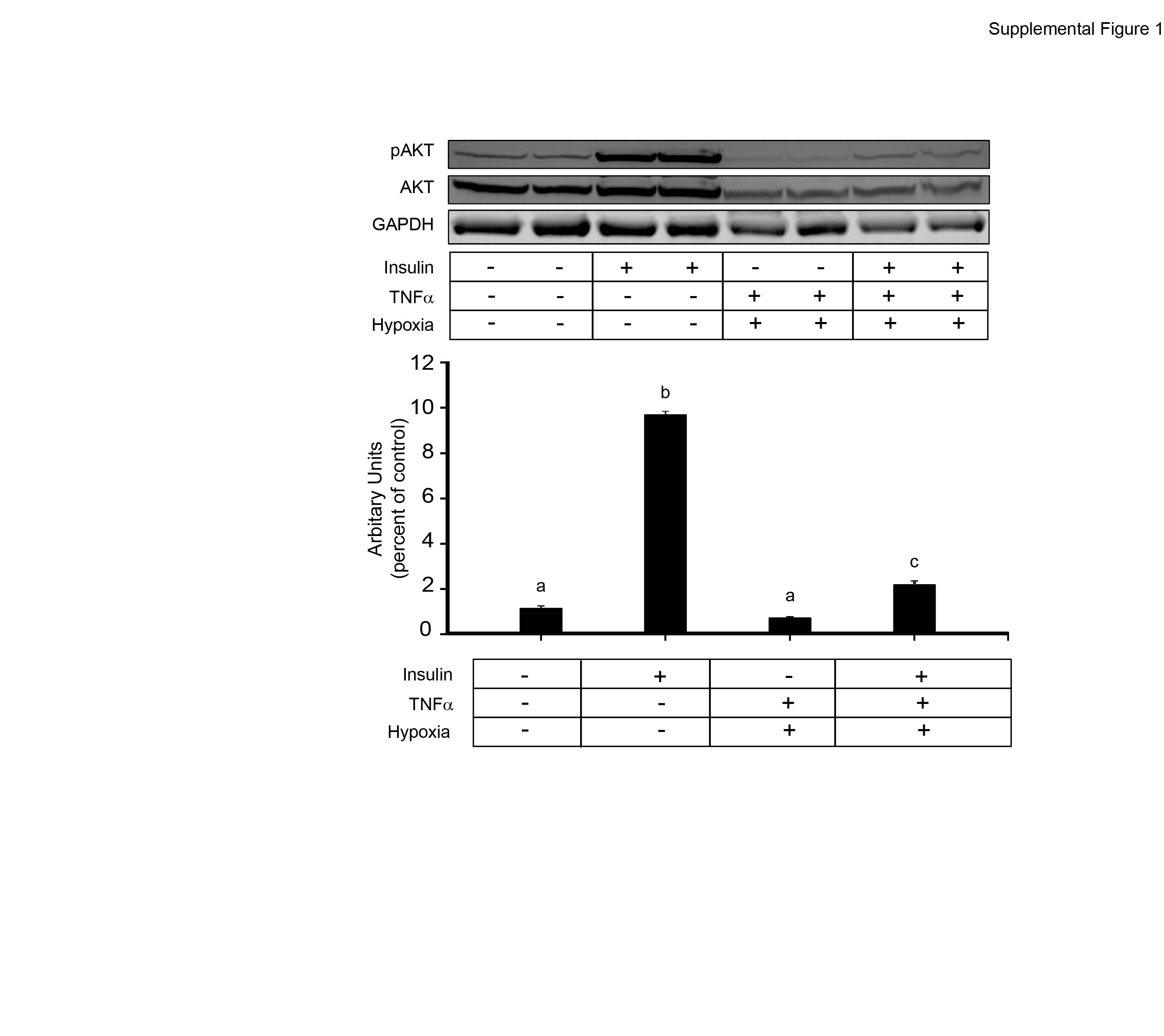

Supplement: Supplemental Material [file KADI_A_2414919_SM0933.zip › Suppl figure 1.jpg]

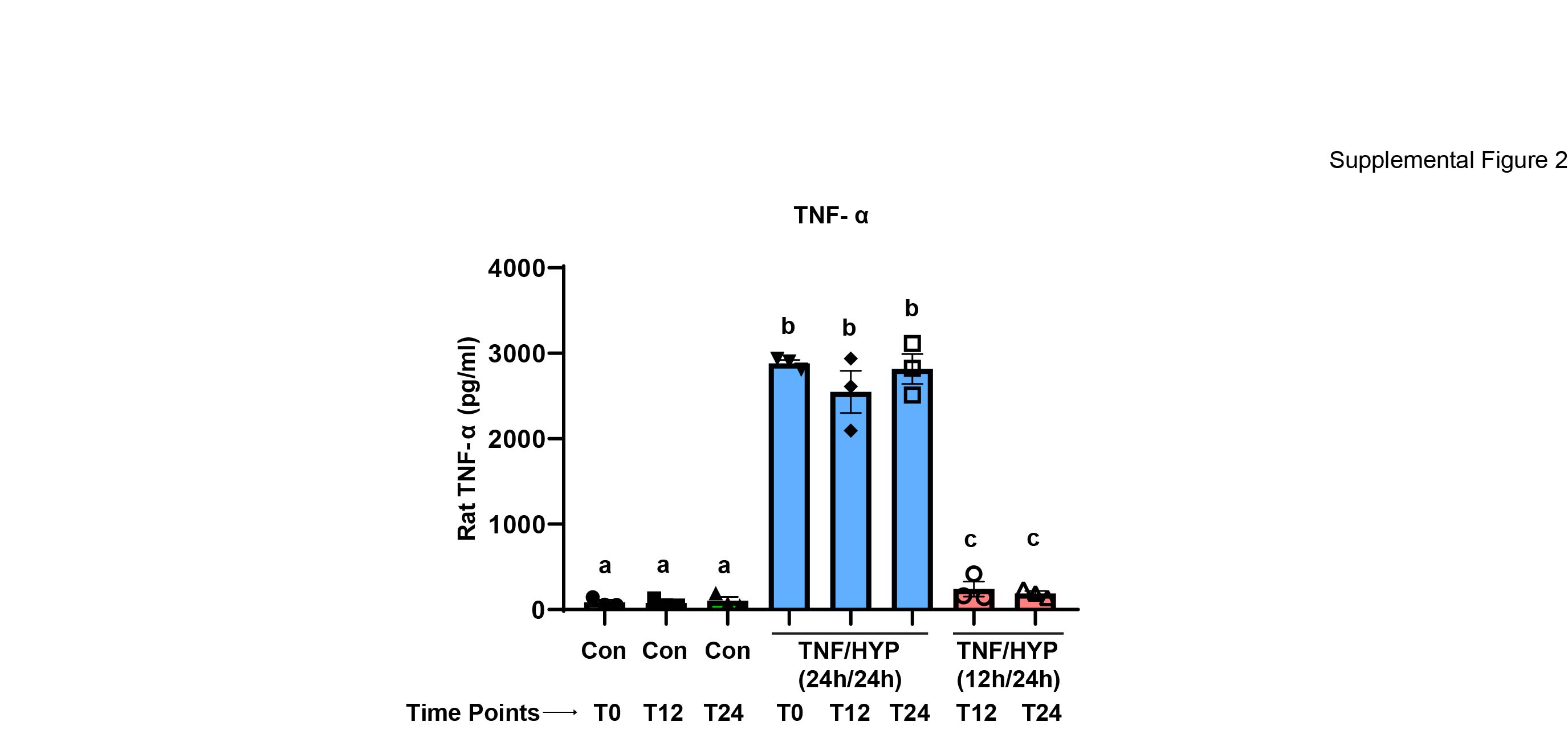

Supplement: Supplemental Material [file KADI_A_2414919_SM0933.zip › Suppl figure 2.jpg]
